# MENTAL HEALTH AND WORK ABILITY OF FEMALE KINDERGARTEN TEACHERS IN GERMANY AND UKRAINE AND THEIR RELATIONSHIP WITH INDIVIDUAL WORK-RELATED BEHAVIORS

**DOI:** 10.13075/ijomeh.1896.02606

**Published:** 2025

**Authors:** Sabine Darius, Angela Sommer, Maryna Lysak, Igor Zavgorodnii, Irina Böckelmann

**Affiliations:** 1 Otto-von-Guericke-University, Department of Occupational Medicine, Magdeburg, Germany; 2 Kharkiv National Medical University, Department of Hygiene and Ecology, Kharkiv, Ukraine

**Keywords:** work ability, kindergarten teachers, mental health, AVEM pattern, subjective stress, country comparison

## Abstract

**Objectives:**

People face stressful situations in different ways and exhibit different work-related behaviors and experiences that can be assigned to a pattern (*Arbeitsbezogenes Verhaltens- und Erlebensmuster* – AVEM). The aim of the study was to determine the mental health and work ability of female kindergarten teachers and their relationship to their individual AVEM patterns.

**Material and Methods:**

In the cross-sectional study, 185 German teachers (D) and 107 Ukrainian teachers (UA) filled out AVEM questionnaire. Mental health and work ability were recorded using questionnaires. Group comparisons were carried out between the kindergarten teachers in both countries.

**Results:**

A total of 126 German and 83 Ukrainian kindergarten teachers could be clearly assigned to 1 of the 4 AVEM patterns: A – effort (18.3% G vs. 38.6% UA), B – burnout (24.6% D vs. 24.1% UA), G – health (17.5% D vs. 25.3% UA) and S – protection (39.7% D vs. 12.0% UA, p < 0.001). German kindergarten teachers rated their work ability (mean [M] ± standard deviation [SD] 7.3±1.7 pts) lower than Ukrainian kindergarten teachers (M±SD 8.0±1.4 pts, p < 0.001). Both groups cope equally well with physical demands, but Ukrainian teachers cope better with mental demands (M±SD 3.7±0.7 pts vs. 3.4±0.8 pts, p = 0.005). Mental health was subjectively impaired in 16.7% of German and 9.6% of Ukrainian kindergarten teachers (p = 0.160). Overall, German teachers rated their mental health worse with M±SD 11.8±5.3 pts (*General Health Questionnaire* total score) than their Ukrainian colleagues with M±SD 8.9±4.6 pts (p < 0.001).

**Conclusions:**

The analysis of individual AVEM patterns can be a helpful basis for identifying health-endangering patterns as well as resources and thus establishing measures to maintain the health of teachers.

## Highlights

- Health-threatening work-related behavioral and experience patterns (*Arbeitsbezogenes Verhaltens- und Erlebensmuster* – AVEM) are associated with reduced work ability.
- Health-threatening AVEM patterns are associated with poor mental health.
- Individual AVEM patterns can complement the assessment of working conditions.
- Preventive measures should be established in the day care centers.

## INTRODUCTION

Kindergarten teachers are exposed to a variety of stresses that can lead to health problems and reduced work ability [1,2]. Serious stress factors include noise [3] and associated high vocal strain [4]. A critical shortage of staff [5] and large groups of children under care [6] place additional burdens on employees. In addition, work tasks must be completed under time pressure [7,8] and negatively experienced communication [9]. Owing to these numerous physical and psychological stressors, kindergarten teachers are at high risk of developing burnout syndrome [7,10]. According to Jungbauer und Ehlen [7], almost 20% of kindergarten teachers are at risk of burnout.

Personal and environmental resources can reduce the effects of stressors on mental health. The importance of work resources in the development of burnout is illustrated by the job-demand-resources model [11]. According to this model, excessive work demands – as aspects of work that cause long-term tension – lead to exhaustion. Too few or inadequate work resources – working conditions that are necessary to achieve goals – reduce work engagement. According to this model, work engagement is seen as the opposite of burnout. It can be increased through appropriate work resources such as support, reward or feedback [12]. In addition to work commitment, important factors include how demands are met, what psychological resilience is used, and work-associated emotions. If a person exerts themselves heavily and their ability to cope leads to subjectively assessed successes, there is hardly any risk to their health. In contrast, if resources are depleted, failure may occur despite a high level of work commitment, posing a health risk [13].

Personal resources can reduce the effects of stressors and thus strengthen mental health and work ability. Personal resources include, for example, relatively stable behavioral and experiential characteristics that describe how the affected person deals with work-related demands and how they process work-related stress. Schaarschmidt and Fischer [13] understand these work-related behavioral and experience patterns (*Arbeitsbezogenes Verhaltens- und Erlebensmuster* – AVEM) in coping with professional demands and work tasks as indicators of the risk of endangering mental health. The authors refer to various concepts described in the literature, such as the concept of coherence experience by Antonovsky [14] or the transactional stress concept according to Lazarus and Folkman [15]. Recognizing these AVEM patterns in a person can help improve their individual resources [16]. Furthermore, belonging to an AVEM pattern or a tendency toward a risk pattern can be interpreted as an indicator of the development of burnout symptoms. As a preventive screening tool, preventive interventions can be initiated without necessarily resulting in a full-blown burnout with sickness-related absences [17]. Previous studies have shown that German educators subjectively rate their mental health lower than Ukrainian educators and also subjectively rate their ability to work worse [2]. In a comparative survey of German and Ukrainian school teachers, no significant differences in work ability were found. However, German teachers were less able to recover and showed a lower ability to relax with increasing age [18].

The study aims to detect differences in these work-related behavior and experience patterns between German and Ukrainian kindergarten teachers and to understand the associations between these AVEM patterns and work ability and mental health. Strengthening individual resources through improving AVEM patterns could maintain work ability and mental health in the context of behavioral prevention. The aim of the study was to find out whether and to what extent the mental health and work ability of German and Ukrainian kindergarten teachers are related to individual work-related behaviours and what effect the AVEM patterns and country affiliation have on these investigated psychological variables.

The following research questions are to be answered:

– Does the distribution of the individual AVEM patterns of the German kindergarten teachers differ from those of the Ukrainian teachers?
– Do the mental health and work ability of kindergarten teachers in both countries differ?
– How are the individual AVEM dimensions related to work ability and mental health?
– What effect do the AVEM patterns and country affiliation have when comparing work ability and mental health?

The results will be used to discuss suggestions for country-specific prevention measures.

## MATERIAL AND METHODS

For the German survey sample, public and private providers of various daycare centers in Magdeburg and the surrounding area were written to request support. Eleven of the 24 providers contacted expressed interest and 28 of a total of 135 facilities participated in the study, carried out in May 2017 – April 2019. The recruitment of Ukrainian kindergarten teachers was carried out in a similar way. The survey of this subsample was carried out between September 2021 – December 2021. The survey was completed 2 months before the start of the war. A positive vote from the Ethics Committee is available for both samples (positive votes from the Ethics Committee of the Otto-von-Guericke-University, Magdeburg, Germany, at the Medical Faculty with registration No. 40/17 and from the Committee for Ethics and Bioethics of the Kharkiv National Medical University [excerpt from the protocol 4/22/7/12, Supplement]).

### Participants

A total of 185 German kindergarten teachers and 107 Ukrainian kindergarten teachers voluntarily participated in the cross-sectional study. The inclusion criteria were voluntary participation in the survey and at least 1 year of employment as a kindergarten teacher. The exclusion criterion was incomplete questionnaire responses. Since only women participated in the study in Ukraine, men in the German sample were not included in the statistical analysis. A further exclusion criterion was non-assignment to an AVEM pattern.

**Figure 1.**
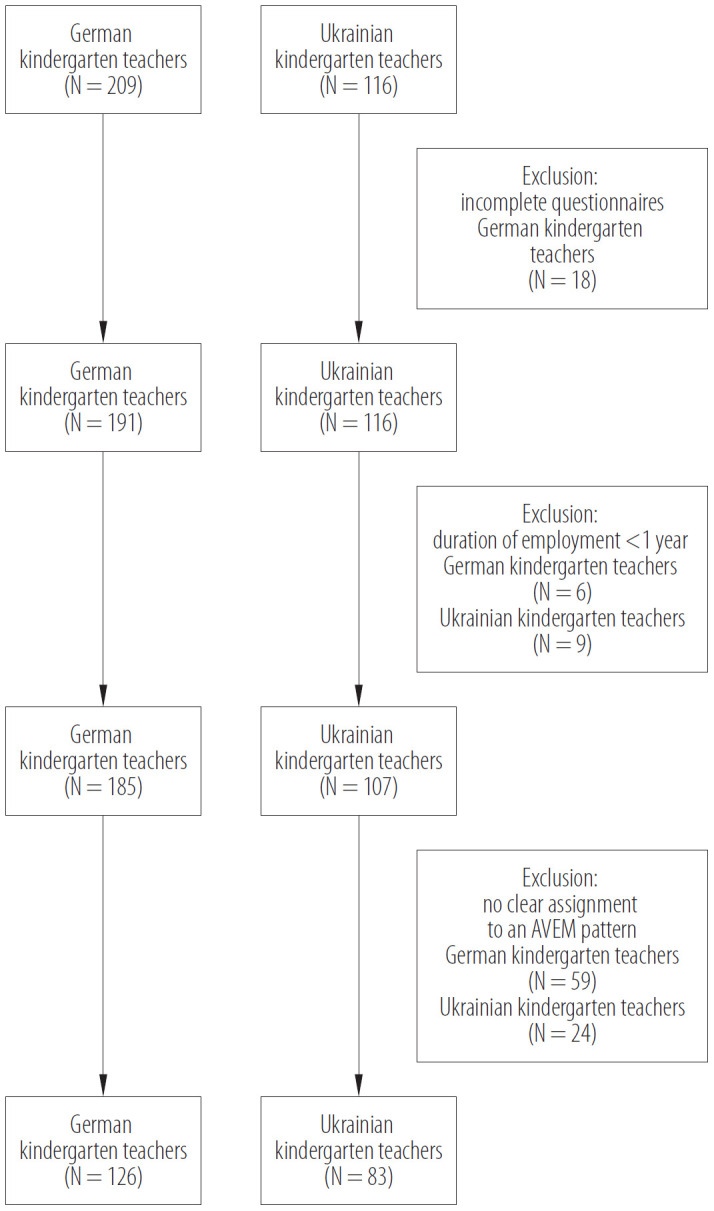
Description of the recruitment process for the study on the mental health and work ability of female kindergarten teachers and their relationship to their individual AVEM patterns, May 2017 – April 2019 (Germany) and September 2021 – December 2021 (Ukraine)

The statistical analysis included only the data of 126 German and 83 Ukrainian female kindergarten teachers who could be clearly assigned to 1 of the 4 AVEM patterns (Figure 1).

### Questionnaires

Sociodemographic information on age group, gender, and professional experience and working time was collected at the beginning of each study using a self-developed questionnaire. Job-related parameters such as the proportion of administrative activities were also measured.

### Work-related behavior and experience patterns

To record AVEM patterns the questionnaire by Schaarschmidt and Fischer [13] was used. The questionnaire contains 66 items with different variants for dealing with stress and questions about attitudes toward work. The answer options are differentiated on a 5-point scale from “completely applies” to “partly” to “does not apply at all.” Six items are assigned to 1 of 11 dimensions. Depending on the answers given and the characteristics of the AVEM dimensions, statements can be made about health-promoting or dangerous attitudes and habits when dealing with work tasks. A distinction is made between these 4 work-related patterns: first, there are 2 healthy patterns: G for “health” and S for “protection,” and second, there are 2 risky patterns: A for “effort” and B for “burnout.”

The G pattern is characterized by high work commitment and high professional ambition while maintaining the ability to distance oneself and resilience to stress. This is achieved by a low tendency to resign in the face of failure coupled with a high level of proactive problem-solving and inner calm and balance.

The S pattern, on the other hand, is characterized by low work-related ambition and a lower willingness to work until exhausted while also being highly satisfied with life. The distancing ability from work is pronounced.

The risk patterns must be distinguished from this: risk pattern A is characterized by very strong manifestations of the dimensions “work-related ambition,” “subjective importance of work,” “willingness to work until exhaust” and “striving for perfection,” with only a very weak distancing ability. Positive dimensions such as “satisfaction with life,” “inner calm and balance” and “experience of social support” are only very weakly pronounced in this risk pattern. Risk pattern B is dominated by low work-related ambition, low willingness to work until exhausted and a very high tendency to resign in the face of failure.

The evaluation was carried out using the Vienna Test System (Fa. Schuhfried, Mödling, Austria), used, among other things, to calculate the probability of belonging to a pattern. If the probability of belonging is >95%, the corresponding pattern is present in its full form, and if the probability is 80–95%, it is present in an accentuated form. The respective pattern tends to be pronounced with a probability 50–80% (with no other pattern >30%). Only those kindergarten teachers who had AVEM patterns according to full, accentuated or tendential expression were included in this study. Kindergarten teachers with a combined expression or with no possibility of assignment were not considered in further evaluation of the data. Cronbach's α as a measure of reliability was 0.504.

### Subjective work ability

Subjective work ability was assessed using 3 questions from the *Work Ability Index* (WAI) [19]. The first item was intended to subjectively assess current work ability, with 0 being defined as “completely unable to work” and 10 as “currently the best work ability.” Two further questions reflected the ability to cope with current work demands, with a distinction being made between coping with physical and psychological demands. The 5 possible answers varied from “very good” (5 pts) to “very bad” (1 pt). Cronbach's α as a measure of reliability was 0.681.

### Mental health

To assess mental health, the *General Health Questionnaire-12* (GHQ-12) was used, in the German translation according to Linden et al. [20]. In this context, the questionnaire serves as a screening instrument for the presence of a mental disorder, but without any claim to diagnosis [21]. Symptoms that could be associated with mental health problems were asked about over the last 4 weeks. For example, questions were asked about whether sleep or concentration problems occurred and whether a lack of self-confidence or feelings of depression or worthlessness played a role. Some of the questions are formulated positively, some negatively, and the answer options are on 4 levels: from “no, not at all” to “as usual” to “much more/less/harder/worse than usual.” Depending on the answer, 0–3 pts are awarded, with a maximum of 36 pts possible, which indicates very poor mental health. In the subsequent dichotomous evaluation of the questionnaire the 2 “positive” answer options are awarded 0 pts and the last 2 “negative” answer options are awarded 1 pt. A maximum of 12 pts can be achieved. In accordance with Üstün et al. [22], Bauer et al. [23] and Seibt et al. [24], a cutoff value for the total GHQ score of ≥5 pts was considered “mentally impaired.” Cronbach's α as a measure of reliability was 0.884.

### Statistical analysis

The statistical evaluation of the raw data was carried out using SPSS software, v. 26 (IBM, Armonk, NY, USA). First, descriptive analyses and tests for a normal distribution were carried out using the Kolmogorov-Smirnov test. Differences in categorical data were calculated using the χ^2^ test or Fisher's exact test. For ordinal scaled data, the Mann-Whitney U test was used to compare German and Ukrainian kindergarten teachers. Statistical differences between kindergarten teachers with different AVEM patterns were tested using the Kruskall-Wallis test. To test the relationships between AVEM patterns and work ability and mental health a partial correlation analysis was carried out with country of origin as a control variable. For the strength of the influence of the AVEM patterns on work ability and mental health with country affiliation as a covariate, a covariance analysis was carried out according to the general linear model. The effect sizes are expressed as partial η^2^, where the effect sizes are calculated according to Cohen [25]: η^2^ <0.06 small effect, η^2^ <0.14 medium effect and η^2^ >0.14 strong effect. A significance level of 5% is used as the basis for all test procedures.

## RESULTS

### Sociodemographic data

Among the participants in the survey, 126 German and 83 Ukrainian kindergarten teachers could be assigned to 1 of the 4 AVEM patterns according to full, accentuated or tendential expression. The distribution of age groups in both samples is shown in Table 1. In the group of <35-year-olds there were more German than Ukrainian kindergarten teachers, and in the group of >45-year-olds – more Ukrainian kindergarten teachers (p = 0.040). Ukrainian kindergarten teachers were more likely to have a university degree (p < 0.001). Many German kindergarten teachers worked part-time, while Ukrainian educators worked full-time, with 1 exception (p < 0.001).

**Table 1.** Socio-demographic and professional data of kindergarten teachers in a country comparison based on the survey carried out in May 2017 – April 2019 (Germany) and September 2021 – December 2021 (Ukraine)

Variable	Participants (N = 209)		p
	Germany	Ukraine	
Age [n (%)]			0.040
<35 years	48 (38.1)	18 (21.7)	
35–45 years	17 (13.5)	16 (19.3)	
≥46 years	61 (48.4)	49 (59.0)	
Education [n (%)]			<0.001
technical school	85 (68.0)	13 (15.7)	
college of applied sciences	27 (21.6)	7 (8.4)	
teacher training college	8 (6.4)	54 (65.1)	
university	5 (4.0)	9 (10.8)	
Working hours [n (%)]			<0.001
full-time	66 (52.4)	82 (98.8)	
part-time	60 (47.6)	1 (1.2)	
Age of children [n (%)]			<0.001
0–3 years	36 (28.6)	14 (16.9)	
4–6 years	34 (27.0)	64 (77.1)	
0–6 years	56 (44.4)	5 (6.0)	
Number of children per group [n (%)]			0.035
<15	18 (14.3)	5 (6.0)	
15–20	60 (47.6)	33 (39.8)	
>20	48 (38.1)	45 (54.2)	
Duration of employment [years]			0.196
M±SD	22.8±13.7	25.6±12.3	
Me (min.–max)	25.5 (2–46)	27 (2–47)	

There was no difference between the 2 samples in terms of length of employment: kindergarten teachers in both countries had worked in the profession for a median of >25 years.

### Work ability, ability to cope with physical and mental demands and mental health

Table 2 compared the subjective assessment of current work ability and self-assessed mental health across countries.

**Table 2.** Assessment of current work ability and coping with physical and mental work demands and distribution of work-related behavior and experience patterns in a country comparison based on the survey carried out in May 2017 – April 2019 (Germany) and September 2021 – December 2021 (Ukraine)

Variable	Germany	Ukraine	p	
			Mann-Whitney U	χ^2^
Subjective current work ability [pts]			**0.021**	
M±SD	7.3±1.80	7.9±1.5		
Me (min.–max)	8.0 (1–10)	8.0 (3–10)		
Coping with physical demands [pts]			0.579	
M±SD	3.8±0.76	3.8±0.71		
Me (min.–max)	4.0 (2–5)	4.0 (2–5)		
Coping with mental demands [pts]			**0.014**	
M±SD	3.4±0.82	3.7±0.72		
Me (min.–max)	3.0 (1–5)	4.0 (2–5)		
Mental health			**<0.001**	
pts				
M±SD	11.8±5.33	8.9±4.59		
Me (min.–max)	11 (4–33)	7 (3–25)		
n (%)				0.150
stable	105 (83.3)	75 (90.4)		
impaired	21 (16.7)	8 (9.6)		
AVEM pattern [n (%)]				**<0.001**
A	23 (18.3)	32 (38.6)		
B	31 (24.6)	20 (24.1)		
G	22 (17.5)	21 (25.3)		
S	50 (39.7)	10 (12.0)		

Bolded are significant differences.

Risk pattern: A – excessive effort, B – burnout, G – health, S – saving.

The current work ability was rated worse by the German kindergarten teachers than by the Ukrainian participants (p = 0.021). In addition, a wider range (1–10 pts) was reported in the German sample than in the Ukrainian sample (3–10 pts). This means that there were test subjects in Germany who considered themselves to be almost completely unable to work with a score of 1 pt. There were no significant differences in the country comparison with respect to the assessment of how well the physical demands were coped with (p = 0.579). On average, German kindergarten teachers rated their ability to cope with the mental demands of work as “average,” whereas their Ukrainian colleagues rated it as “rather good” (p = 0.014).

### Mental health

German kindergarten teachers rated their mental health worse than their Ukrainian colleagues (p < 0.001) (Table 2). After applying the cutoff value of ≥5 pts to classify mental health as stable vs. impaired, the differences found between the kindergarten teachers in both countries disappeared. A total of 16.7% of the German kindergarten teachers and 9.6% of the Ukrainian kindergarten teachers had impaired mental health (p = 0.150) (Table 2).

### Work-related behavior and experience patterns

There were significant differences between the 2 countries with respect to categorization into AVEM patterns (Table 2). After the questionnaire was analyzed, the teachers were divided into the following patterns: A – effort (18.3% D vs. 38.6% UA), B – burnout (24.6% D vs. 24.1% UA), G – health (17.5% D vs. 25.3% UA) and S – relaxation (39.7% D vs. 12.0% UA, p <0.001).

**Figure 2.**
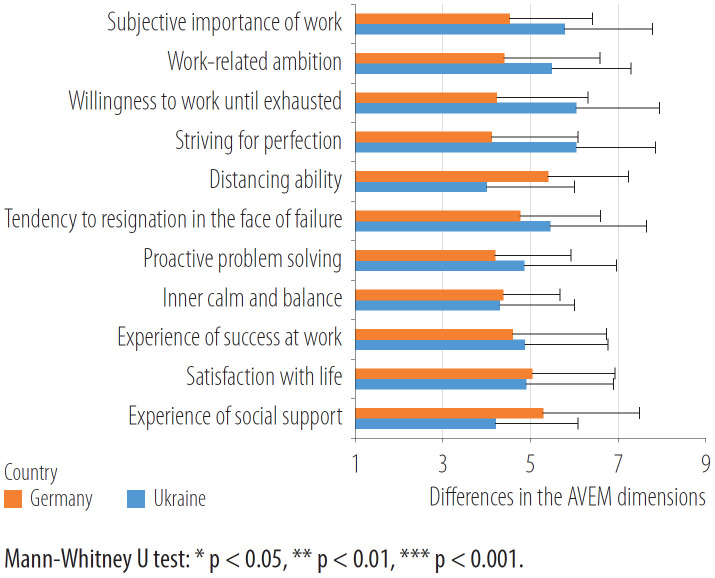
Differences in the work-related behavioral and experiential dimensions between the kindergarten teachers in both countries based on the survey carried out in May 2017 – April 2019 (Germany) and September 2021 – December 2021 (Ukraine)

The differences in the individual AVEM dimensions are shown in Figure 2.

In the German sample, the dimensions “subjective importance of work,” “work-related ambition,” “willingness to work until exhaust,” “striving for perfection” and “tendency to resign in face to failure” are statistically significantly lower, and “distancing ability” and “experience of social support” are more pronounced (p < 0.05) than in the Ukrainian sample.

### Subjective current work ability and coping with work demands in the context of AVEM patterns

The self-assessed work ability differs both within the AVEM patterns and within the respective groups between German and Ukrainian kindergarten teachers (Table 3). German kindergarten teachers with risk pattern B, in particular, rate their current work ability as low compared with both Ukrainian kindergarten teachers with the same pattern (p = 0.024) and German kindergarten teachers with health-promoting patterns G (p < 0.001) and S (p = 0.013).

**Table 3.** Self-assessed work ability and coping with work demands as a function of work-related behavior and experience patterns (*Arbeitsbezogenes Verhaltens- und Erlebensmuster* – AVEM) in a country comparison based on the survey carried out in May 2017 – April 2019 (Germany) and September 2021 – December 2021 (Ukraine)

Variable	Subjective current work ability					Coping with physical demands					Coping with mental demands				
	Germany	p	Ukraine	p	p^c^	Germany	p	Ukraine	p	p^c^	Germany	p	Ukraine	p	p^c^
AVEM pattern		**<0.001**^a^, B–S **0.013**^b^, B–G **<0.001**^b^		0.056^a^			**0.005**^a^, B–G **0.013** ^b^		0.071^a^			**0.003**^a^, B–G **0.007**^b^		**<0.001**^a^, B–A **0.009**^b^, B-G **<0.001**^b^	
A					0.719					0.323					0.164
M±SD [pts]	7.6±1.6		7.8±1.6			3.6±0.6		3.9±0.7			3.4±0.8		3.8±0.6		
Me (min.–max) [pts]	8 (3–10)		8 (3–10)			4 (2–5)		4 (3–5)			4 (2–5)		4 (3–5)		
B					**0.024**					0.703					0.924
M±SD [pts]	5.9±2.2		7.5±1.8			3.4±0.9		3.5±0.7			2.9±0.9		3.0±0.6		
Me (min.–max) [pts]	6 (1–9)		7.5 (5–10)			3 (2–5)		3.5 (2–5)			3 (1–4)		3 (2–4)		
G					0.161					0.687					0.155
M±SD [pts]	8.3±0.8		8.7±1.1			4.1±0.7		4.1±0.7			3.8±0.8		4.1±0.6		
Me (min.–max) [pts]	8 (7–9)		9 (6–10)			4 (3–5)		4 (3–5)			4 (2–5)		4 (3–5)		
S					0.728					0.808					0.115
M±SD [pts]	7.6±1.4		7.8±1.2			3.9±0.7		3.9±0.6			3.4±0.6		3.8±0.6		
Me (min.–max) [pts]	8 (5–10)		8 (5–9)			4 (2–5)		4 (3–5)			3 (2–5)		4 (3–5)		

Risk patterns: A – excessive effort, B – burnout, G – health, S – saving.

Bolded are significant differences.

a Kruskal-Wallis p.

b Bonferroni (adjusted significance values after Bonferroni correction for multiple tests) p.

c Mann-Whitney U p.

Further differences between the kindergarten teachers in the country comparison were not statistically significant. Differences were found only between the AVEM patterns: German kindergarten teachers with pattern B are less able to cope with physical and mental demands than kindergarten teachers with pattern G are (p = 0.024 and p = 0.007, respectively), and they generally rate their work ability significantly worse than kindergarten teachers with patterns G (p = 0.013) and S (p < 0.001). Compared with Ukrainian kindergarten teachers with patterns G (p < 0.001) and A (p = 0.009), Ukrainian kindergarten teachers with pattern B cope less well with psychological demands.

### Mental health in the context of AVEM patterns

**Figure 3.**
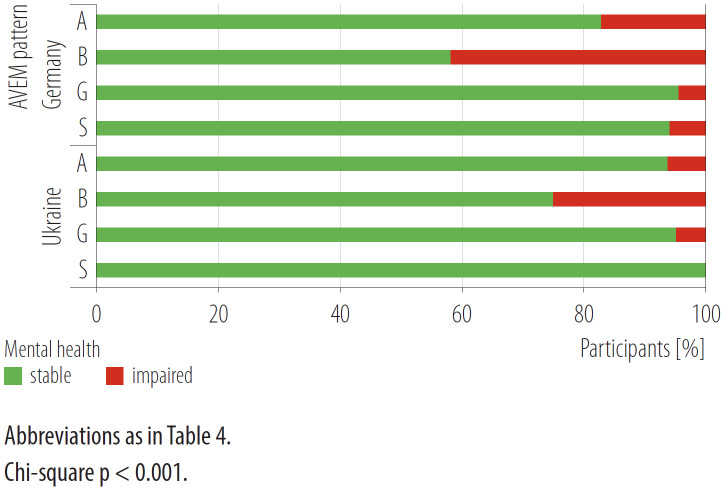
Distribution of stable and impaired mental health depending on the work-related behavior and experience pattern (*Arbeitsbezogenes Verhaltens- und Erlebensmuster* – AVEM) in a country comparison based on the survey carried out in May 2017 – April 2019 (Germany) and September 2021 – December 2021 (Ukraine)

Highly significant differences in mental health were found in the distribution of the 4 AVEM patterns across the GHQ groups (Figure 3). The mental health of kindergarten teachers with a health-endangering pattern affiliation (patterns A and B) is more likely to be impaired (p < 0.001).

In particular, kindergarten teachers with pattern B rate their mental health significantly worse than kindergarten teachers with health-promoting patterns G and S. The mental health of all Ukrainian kindergarten teachers with pattern S is stable. With respect to the evaluation of mental health after classification into AVEM patterns, it was found that in each of the patterns the German kindergarten teachers achieved higher mean values, which corresponds to poorer mental health (Table 4). Among the AVEM patterns, mental health differs across countries (p < 0.001). However, in the individual comparisons there were only differences in pattern A, where the German kindergarten teachers reported poorer mental health (p < 0.001).

**Table 4.** Mental health as a function of work-related behavior and experience patterns (*Arbeitsbezogenes Verhaltens- und Erlebensmuster* – AVEM) affiliation in a country comparison based on the survey carried out in May 2017 – April 2019 (Germany) and September 2021 – December 2021 (Ukraine)

Variable	Mental health						p
	Germany			Ukraine			
	M±SD [pts]	Me (min.–max) [pts]	p	M±SD [pts]	Me (min.–max) [pts]	p	
AVEM pattern			**<0.001**^a^, G–B **<0.001**^c^, S–A **0.012**^c^, S–B **<0.001**^c^			**<0.001**^a^, G–B **0.002**^c^, A–B **0.020**^c^	**<0.001**^a^, **<0.001**^b^
A	13.2±5.35	12.0 (5–29)		8.2±4.27	6.6 (3–21)		
B	16.1±6.39	15.0 (5–33)		12.5±5.37	11.0 (5–25)		
G	8.8±2.85	8.5 (4–17)		6.9±3.12	6.0 (3–16)		
S	9.8±3.23	10.0 (5–21)		8.3±2.54	7.5 (5–13)		

Bolded are significant differences.

Risk patterns: A – excessive effort, B – burnout, G – health, S – saving.

a Kruskal-Wallis p.

b Bonferroni p.

c Bonferroni (adjusted significance values after Bonferroni correction for multiple tests) p.

There were differences in mental health among kindergarten teachers with pattern A in the country comparison (13.2 pts D vs. 8.2 UA, p < 0.001). Compared with Ukrainian kindergarten teachers, remarkably high mean values of 16.1 pts were achieved in Germany within AVEM pattern B, with 12.5 pts. Among the AVEM patterns in the respective countries Ukrainian kindergarten teachers with pattern B had poorer mental health than kindergarten teachers with pattern G (p = 0.002) and pattern A (p = 0.020). The mental health of German kindergarten teachers with health-promoting pattern G was better than that of nursery teachers with health-endangering pattern B (p < 0.001). Kindergarten teachers with pattern S had better mental health than kindergarten teachers with health-endangering patterns A (p = 0.012) and B (p < 0.001).

### Correlation analyses

To identify correlations between the individual AVEM dimensions and work ability and mental health (here, the total GHQ score), a partial correlation analysis was carried out, controlling for nationality (Table 5).

**Table 5.** Relationships between the work-related behavior and experience patterns (*Arbeitsbezogenes Verhaltens- und Erlebensmuster* – AVEM) dimensions, work ability, and mental health based on the survey carried out in May 2017 – April 2019 (Germany) and September 2021 – December 2021 (Ukraine)

AVEM dimension	Variable			
	subjective current work ability	coping with physical demands	coping with mental demands	mental health
Subjective importance of work	**0.183** **	0.131	**0.220** **	–0.086
Work-related ambition	**0.308** ***	**0.182** **	**0.172** *	–0.079
Willingness to work until exhausted	–0.038	–0.094	–0.076	**0.265** ***
Striving for perfection	**0.232** ***	**0.145** *	**0.155** *	0.088
Distancing ability	**0.190** **	**0.235** ***	**0.255** ***	**–0.348** ***
Tendency to resignation in the face of failure	–0.128	–0.116	**–0.251** ***	**0.424** ***
Proactive problem-solving	**0.255** ***	**0.249** ***	**0.368** ***	**–0.338** ***
Inner calm and balance	**0.168** *	**0.204** **	**0.233** ***	**–0.231** ***
Experience of success at work	**0.356** ***	**0.172** *	**0.264** ***	**–0.358** ***
Satisfaction with life	**0.385** ***	**0.301** ***	**0.389** ***	**–0.587** ***
Experience of social support	**0.142** *	0.131	**0.202** **	**–0.201** **

Bolded are statistically significant correlations.

* p < 0.05;

** p < 0.01;

*** p < 0.001.

There was a medium correlation between the subjectively assessed work ability and satisfaction with life, experience of success at work and work-related ambition (p < 0.001 in each case). There were medium positive correlations of mental health with the tendency to resign in the face of failure and medium negative correlations with distancing ability, proactive problem-solving ability and the experience of success at work (each p < 0.001). Mental health correlated strongly with satisfaction with life (ρ = –0.587, p < 0.001).

The general linear model was used to examinethe effect of individual AVEM patterns and country affiliation when comparing mental health and work ability (Table 6).

**Table 6.** Effects of the covariates work-related behavior and experience patterns (*Arbeitsbezogenes Verhaltens- und Erlebensmuster* – AVEM) and country affiliation on work ability and mental health based on the survey carried out in May 2017 – April 2019 (Germany) and September 2021 – December 2021 (Ukraine)

Variable	Corrected model			AVEM pattern		Country	
	F	p	η^2^	p	η^2^	p	η^2^
Current work ability	5.182	**0.007**	0.057	**0.043**	0.024	**0.003**	0.050
Mental health	15.133	**<0.001**	0.150	**<0.001**	0.10	**<0.001**	0.110

η^2^ – partial eta^2^: <0.06 small effect, <0.14 medium effect, >0.14 strong effect.

Bolded are statistically significant correlations.

The model explains 5.7% of the variance in work ability and 15% of the variance in mental health. The pattern affiliation according to AVEM and the country affiliation each have a medium effect on mental health and a small effect on work ability. After analyzing the partial η^2^, membership in the individual AVEM patterns explains 10% of mental health (GHQ) and 2.4% of work ability (WAI). The country explains 11% of the variance in mental health and 5% of the variance in work ability.

## DISCUSSION

This study reveals the connection between AVEMs, on the one hand, and subjectively assessed work ability and mental health, on the other hand, for female kindergarten teachers from Germany and Ukraine. German kindergarten teachers are more likely to be assigned to AVEM pattern S (orientated toward protection), while Ukrainian kindergarten teachers increasingly belong to risk pattern A. The GHQ scoring revealed reduced mental health in just <17% of German and 10% of Ukrainian kindergarten teachers. In particular, it is educators with risk pattern B whose ability to work and mental health are limited.

Thielmann et al. [26] reported a similar distribution of AVEM patterns among Ukrainian female teachers. In this sample the majority of participants were assigned to risk type A. A study conducted specifically among German music teachers revealed a predominant allocation to AVEM risk patterns A and B [16]. However, the S pattern was also frequently found among older music teachers. In a survey in North Rhine-Westphalia, Mauz et al. [27] reported that the majority of educational professionals (35%) responded with the S pattern. The majority of the German kindergarten teachers in this sample were assigned to the S pattern, too. Overall, approx. 63% of Ukrainian kindergarten teachers belong to a risk pattern, which is consistent with the results of Thielmann et al. [28] in their survey of Ukrainian university lecturers. In contrast, only approx. 43% of German study participants fall into an AVEM risk pattern. A possible explanation for this discrepancy could be better health education, opportunities for stress prevention, and access to occupational health screenings in Germany [29,30]. As the ability to distance oneself is particularly limited in risk patterns A and B, people whose work-related behavior corresponds to these patterns should be given early preventive intervention. For example, relaxation courses help improve the ability to distance oneself from others [30,31]. Carroll et al. [30] reported that both stress reduction programs and health programs led to improved well-being among teachers, which subsequently also had a positive effect on the students. People who correspond to the AVEM pattern S have a good capacity for detachment and are more likely to have a lack of motivation [32]. Given that a lack of motivation and disinterest in work can also represent a risk for the development of mental illnesses, preventive measures adapted to this type of illness must be determined. For example, a constant lack of motivation can lead to boredom, a “boreout” or even depression [33]. A corresponding preventive measure in this case would be to expand the employees' scope for action to underpin their motivation through self-efficacy.

German kindergarten teachers rate their work ability lower than Ukrainian kindergarten teachers and they also rate their ability to cope with mental work demands lower. For teachers, Thielmann et al. [18] reported only a trend toward slightly lower work ability among German teachers. However, this comparison is difficult, as only the first 3 items of the WAI questionnaire were used in the authors' study. Compared with a study of music school teachers, the kindergarten teachers in this study rated their work ability slightly lower [34]. One possible clue as to why German kindergarten teachers in this study rate their ability to work lower than Ukrainian kindergarten teachers could be related to the generally sharp rise in sickness rates in Germany in recent years [35]. Ukrainian data from the same survey are only available up to 2010, but show significantly lower sickness rates compared to Germany in the same period. Another possible factor could be the legal regulations governing sickness-related absence from work. In Germany, employees receive full continued pay for 6 weeks in the event of illness. In Ukraine, financial reductions begin after the sixth day of illness unless they can prove several years of service.

In an intervention study of kindergarten teachers in Saxony, Seibt et al. [36] reported that kindergarten teachers with limited work ability had a reduced ability to recover. In this study, the ability to switch from work was also correlated with reduced work ability and impaired mental health. A reduced ability to recover can in turn be interpreted as an early indicator of burnout syndrome [31].

In this study, life satisfaction was strongly correlated with mental health. Therefore, the life satisfaction of kindergarten teachers could be strengthened as a further preventive approach. According to a study by Bellé et al. [37], a good feedback culture increased the satisfaction of kindergarten teachers. In a questionnaire survey, Jungbauer and Ehlen [7] reported that social support and teamwork are very important for improving the well-being of kindergarten teacher staff. The stronger the level of support, the greater the level of satisfaction and the lower the rate of depression [38]. Increasing the expectation of self-efficacy or self-esteem can also have a positive influence on satisfaction [39,40]. A high level of job satisfaction increases employee motivation, and kindergarten teachers remain in the job [41,42]. In addition, Faragher et al. [43] reported a strong correlation between job and mental health satisfaction, which also suggests a positive effect of job satisfaction on life satisfaction.

In Ukraine, the profession of kindergarten teacher has been academized. It is possible that Ukrainian kindergarten teachers are more valued as a result and that their social status is recognized. This results in additional resources that keep the kindergarten teachers healthy. The subjectively more stable assessment of mental health by Ukrainian kindergarten teachers may be partly related to the continuing strong stigmatization of mental illness in Ukraine [44]. Another factor may be the lack of awareness and education about mental stress and illness in Ukraine [45].

To strengthen the mental health and thus the work ability of kindergarten teachers 2 starting points are conceivable, namely, situational and behavioral prevention. At the level of behavioral prevention, it can be ensured that more staff are employed in daycare centers to spread the work over several shoulders or to improve the working conditions of employees and thus prevent stress. The promotion of social resources, team development and leadership skills can also contribute to a better situation and therefore a reduction in stress levels among staff [7]. The development and strengthening of different competences can therefore make the work of employees easier. Other preventative measures include the improvement of work processes, improved working time organization and opportunities for self-determination and greater freedom of action for kindergarten teachers. In addition, Shin et al. [46] showed that the behavior of employers toward employees has a significant influence on their work motivation. Specialized communication training at the management level could be used as a preventative measure in this regard.

In a multimodal approach, personal resources can also be strengthened at the individual level (behavioral prevention). In stress management courses, kindergarten teachers can learn more positive stress management strategies. Employees in this occupational group should, in particular, have the skills of conflict management, time management, self-efficacy, empathy and a sense of coherence to be able to deal with professional situations with less stress and thus prevent the development of burnout syndrome. Individual resources can and should be strengthened, for example, by implementing further training programs (relaxation courses, time management, etc.). It is important to take preventative action, especially in the context of demographic changes in the world of work and the increasing aging of employees in this occupational group. Intervention programs tailored to older kindergarten teachers have been shown to strengthen the work ability and maintain the health of kindergarten teachers [47].

Employees with health-threatening patterns A and B should be offered measures to help them break out of these patterns. These include, for example, offering courses on optimizing time management and learning to say “no” in the event of an increased tendency to overextend themselves. A realistic assessment of work tasks should also be communicated by the manager. Relaxation training such as yoga, meditation or progressive muscle relaxation can help with limited distancing skills, as described by Thielmann et al. [16].

In addition to proportional and behavioral prevention as primary prevention measures, both the early detection of burnout as part of secondary prevention (occupational health care) and reintegration into work after a long-term illness as part of tertiary prevention should not be neglected.

The implementation of preventive occupational health care, as is already prescribed in Germany, should also be carried out in Ukrainian facilities to complement the range of services. During the consultation, the company doctor could recognize the favorable influences of personal factors that could have a negative impact on mental health. In particular, low life satisfaction should be identified.

### Limitations

Many providers of kindergartens in Magdeburg and the surrounding area did not respond to the letter advertising the study. This presumably created bias. The situation may be better for the providers who are interested in the study than for the facilities of the providers who ignored the letter. With the support of the organizations, the study could be carried out in the facilities during working hours, which may have led to increased participation by the employees. The kindergarten teachers who took part in the study on their own initiative following word-of-mouth propaganda may have had more limited work ability or poorer mental health than the kindergarten teachers whose providers had supported the study. It can also be assumed that the kindergarten teachers who took part in self-motivation were less satisfied with their profession or employer.

The questionnaire survey was conducted among German kindergarten teachers before the SARS-CoV-2 pandemic, whereas in Ukraine, it took place during the subsequent outbreak period. It is very likely that Ukrainian kindergarten teachers had to cope with additional stress during the pandemic due to numerous infection control measures and cases of illness.

The questionnaires on work ability and mental health were translated and checked by native speakers. No back-translation was performed. This may have led to misunderstandings on the part of the Ukrainian teachers.

The questionnaire survey was also limited to a localized area (Magdeburg and Kharkiv) so it is not possible to draw conclusions about the population of German and Ukrainian kindergarten teachers. For example, there are already considerable differences in the childcare ratio in day care facilities in Germany, with significantly more children being cared for by full-time employees in the eastern German federal states than in the western German federal states.

For data protection reasons, the kindergarten teachers were not asked their specific age. The survey was anonymous. To avoid drawing conclusions about the respective kindergarten teachers from their date of birth (which would be particularly dangerous in smaller facilities), age groups were formed into which kindergarten teachers could categorize themselves. However, this entails a loss of information.

It should be noted that the surveys were completed before the start of the war in Ukraine. The data therefore represents the status before 2022. The authors did not examine the impact of the war on the (mental) health of the kindergarten teachers or on the results. A comparison with the current situation is not possible. If a survey were conducted at this time, the results might be different. However, this is speculation.

## CONCLUSIONS

Health-threatening AVEM patterns are associated with reduced work ability and poorer mental health. Recording individual AVEM patterns can usefully complement the analysis and assessment of working conditions.

Country-specific preventive measures should therefore be established in the centers to promote and maintain the health of kindergarten teachers. For example, it would be helpful to hire more staff to distribute work among several employees. Promoting social resources, team development and leadership skills can also lead to a reduction in stress levels. At an individual level, kindergarten teachers with risk pattern should be offered stress management and relaxation courses. In addition, more mindfulness courses could be established in which participants recognize stressors and learn how to deal with them appropriately. Sporting activities, e.g., as part of prevention courses, can also help people with risk patterns A and B reduce tension and activate the reward system. In contrast, motivating and activating measures can help increase work engagement in the S pattern and prevent “boreout.” The AVEM is also a good measurement tool over time. It can be used regularly, for example, every 2 years, to check whether measures have achieved the desired effects. It can also indicate changes from a G pattern to a risky pattern and can thus be used as an “early warning system.” A trusting relationship with the occupational physician regarding confidentiality with the employer is important here; otherwise, falsifications in the sense of “desired answers” could occur.
